# Photoinduced charge-transfer dynamics in fluorescent electron donor–acceptor polymers

**DOI:** 10.1039/d5sc07237a

**Published:** 2025-11-25

**Authors:** Estefanía Sucre-Rosales, Suiying Ye, Yinyin Bao, Eric Vauthey

**Affiliations:** a Department of Physical Chemistry, University of Geneva 30 Quai Ernest-Ansermet CH-1211 Geneva 4 Switzerland eric.vauthey@unige.ch; b Department of Chemistry and Applied Biosciences, ETH Zurich Vladimir Prelog Weg 1 8093 Zurich Switzerland; c Department of Chemistry, Faculty of Science, University of Helsinki A. I. Virtasen aukio 1 00014 Helsinki Finland yinyin.bao@helsinki.fi

## Abstract

Electron donor–acceptor (D–A) polymers are emerging as promising candidates for the development of solid materials with tunable emission. Herein, we investigate the excited-state dynamics of polymers consisting of a central naphthalenediimide (NDI) acceptor with two polystyrene donor chains and copolymers with various secondary donors incorporated. We find strong differences in the dynamics when going from diluted polymer solutions to pure polymer films. In liquids, ultrafast intrachain electron transfer from a styrenic donor to the excited NDI, followed by sub-nanosecond charge recombination to the ground state is observed. Because of the tight packing in the film, ultrafast electron transfer occurs between donors and acceptors of different polymer chains. Emission is found to originate from the most strongly coupled D–A pairs, for which electron transfer is so fast that it leads to a lifetime broadening of the NDI absorption band. Because of this, these highly coupled pairs can be photoselected upon red-edge excitation. The charge-transfer state decays on the tens of ns timescale *via* radiative and non-radiative charge recombination to the ground state as well as *via* charge recombination to the triplet state of NDI. This latter pathway, which is detrimental to the fluorescence quantum yield, is almost suppressed with the strongest secondary donor. Finally, we show that excitation of the secondary donor instead of the NDI acceptor does not lead to the population of the charge-transfer state and thus does not contribute to the luminescence of the films.

## Abstract 2

Ultrafast charge-separation occurs upon photoexcitation of fluorescent electron donor–acceptor polymer films. The pathways, which compete with radiative recombination, are identified, allowing for possible improvement in fluorescence efficiency.

## Graphical abstract

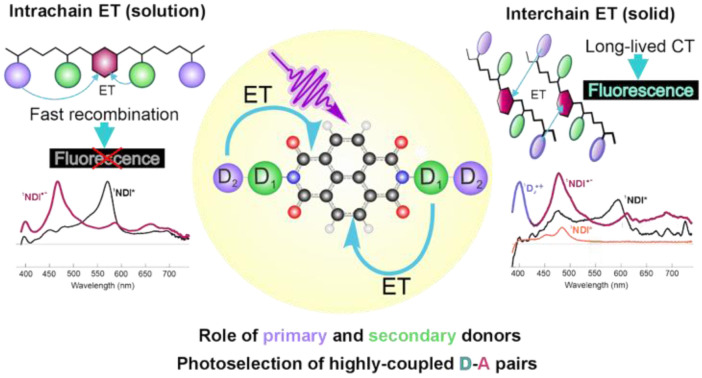

## 1 Introduction

Over the past few years, considerable research effort has been dedicated to the development of light-emitting materials with tunable emission wavelength and quantum yield,^1–4^ especially emissive polymers owing to their solution-processability,^5,6^ and synthetic controllability.^7–9^ Among them, through-space charge transfer (CT) polymers with electron donor and acceptor units incorporated in the polymer backbone or side groups have attracted great attention due to their strong emission^10–12^ and versatile tunability.^13–16^ Highly efficient colour tuning of molecular emission in the solid state was recently demonstrated with a library of styrenic donor (D_1_) polymers grown from a naphthalene diimide (NDI) acceptor (A) by atom-transfer radical polymerisation.^17,18^ Continuous blue to red emission tuning was achieved by combining three different strategies: (i) variation of D_1_, (ii) modification of the polymer end with secondary donor groups, D_2_, and (iii) variation of the polymer chain length. As these polymers are based on a non-conjugated structure, CT emission occurs *via* through-space interactions between A and D_1_ and/or A and D_2_. This emission is, thus, similar to that of exciplexes or of excited D–A complexes.^19–21^ The difference between these two excited species is that an exciplex is formed upon diffusive encounter between a photoexcited A(D) and a D(A) quencher, whereas CT emission from a D–A complex usually results from direct optical excitation in the CT absorption band.^20,22^

**Fig. 1:**
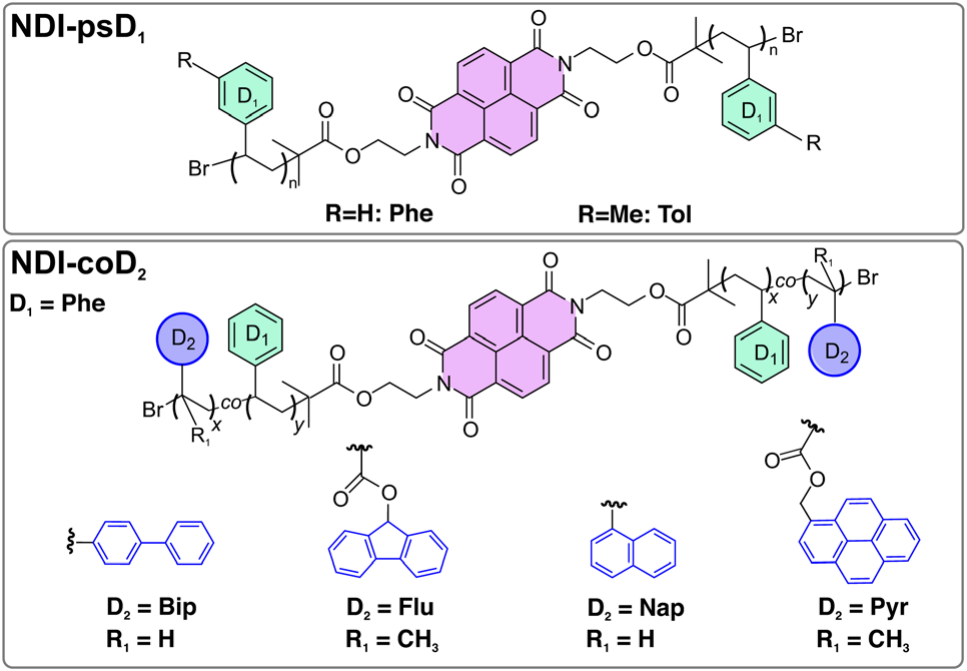
Structures of the NDI-psD_1_ polymers (up) and NDI-coD_2_ copolymers (bottom).

While the excited-state properties of conjugated D–A polymers have been investigated in detail, very little is known about the photophysics of these emissive non-conjugated CT polymers, apart from their basic fluorescence properties.^17^ Understanding of how the emissive state is populated and knowledge of possible competing pathways, which could be detrimental to their properties, are crucial for further development and optimisation of this type of material. Here, we report on our investigation of the excited-state dynamics of two NDI-based D–A polymers with phenyl donors, NDI-psD_1_, as well as four copolymers with secondary end donors of varying strength, NDI-coD_2_ (Fig. 1), using ultrafast transient electronic absorption spectroscopy. We first compare the photoinduced dynamics of liquid solutions of (co)polymers with those of spin-coated films and show that inter-chain interactions are crucial for the occurrence of CT emission. We also demonstrate that CT emission occurs from strongly coupled D–A pairs for which electron transfer (ET) is fast enough to broaden the absorption spectrum of the NDI acceptor. As a consequence, these pairs can be photoselected upon red-edge excitation. Finally, we discuss the behaviour of NDI-coPyr, where both the NDI acceptor and the Pyr secondary donor are photoexcited. We show that, in solution, excitation of the secondary donor opens an additional pathway towards a long-lived CT state. In films, on the other hand, Pyr acts as an inner filter and its excitation does not lead to CT emission. These findings should be very useful for further improvements of the emissive properties of non-conjugated D–A polymers.

## 2 Results and discussion

### 2.1 Liquid solutions *vs.* films

#### 2.1.1 Stationary spectroscopy

**Fig. 2:**
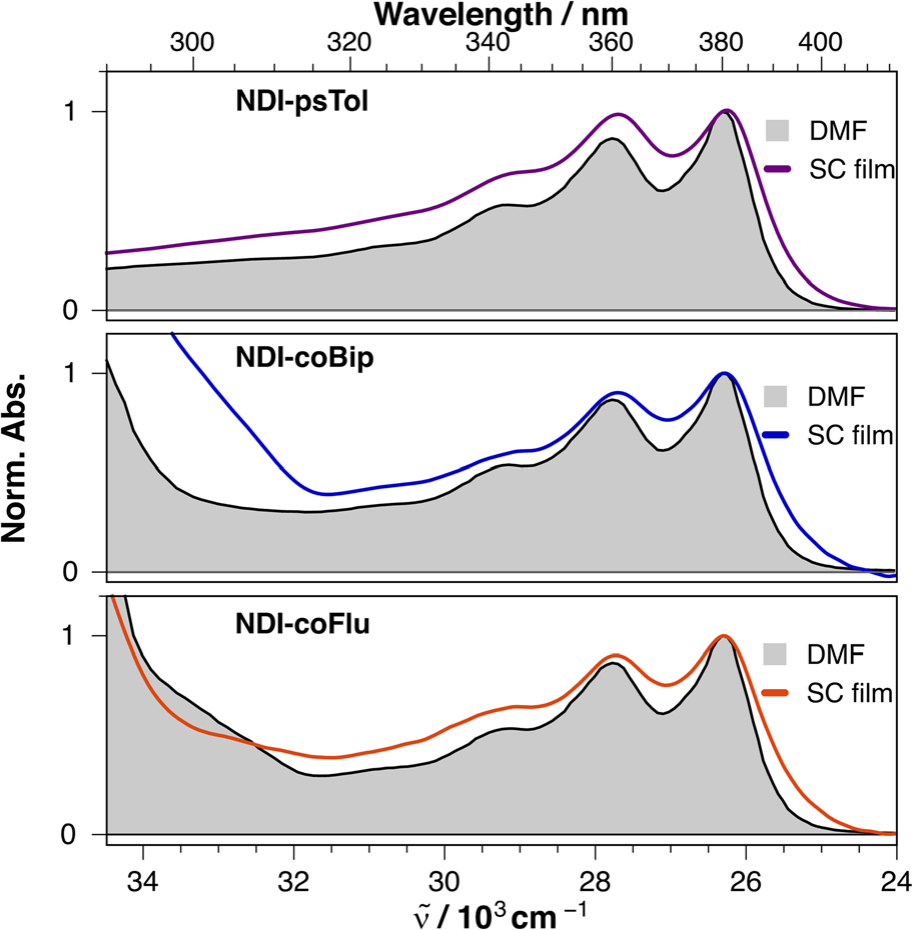
Comparison of the electronic absorption spectra of (co)polymers in DMF solution and films.

**Fig. 3:**
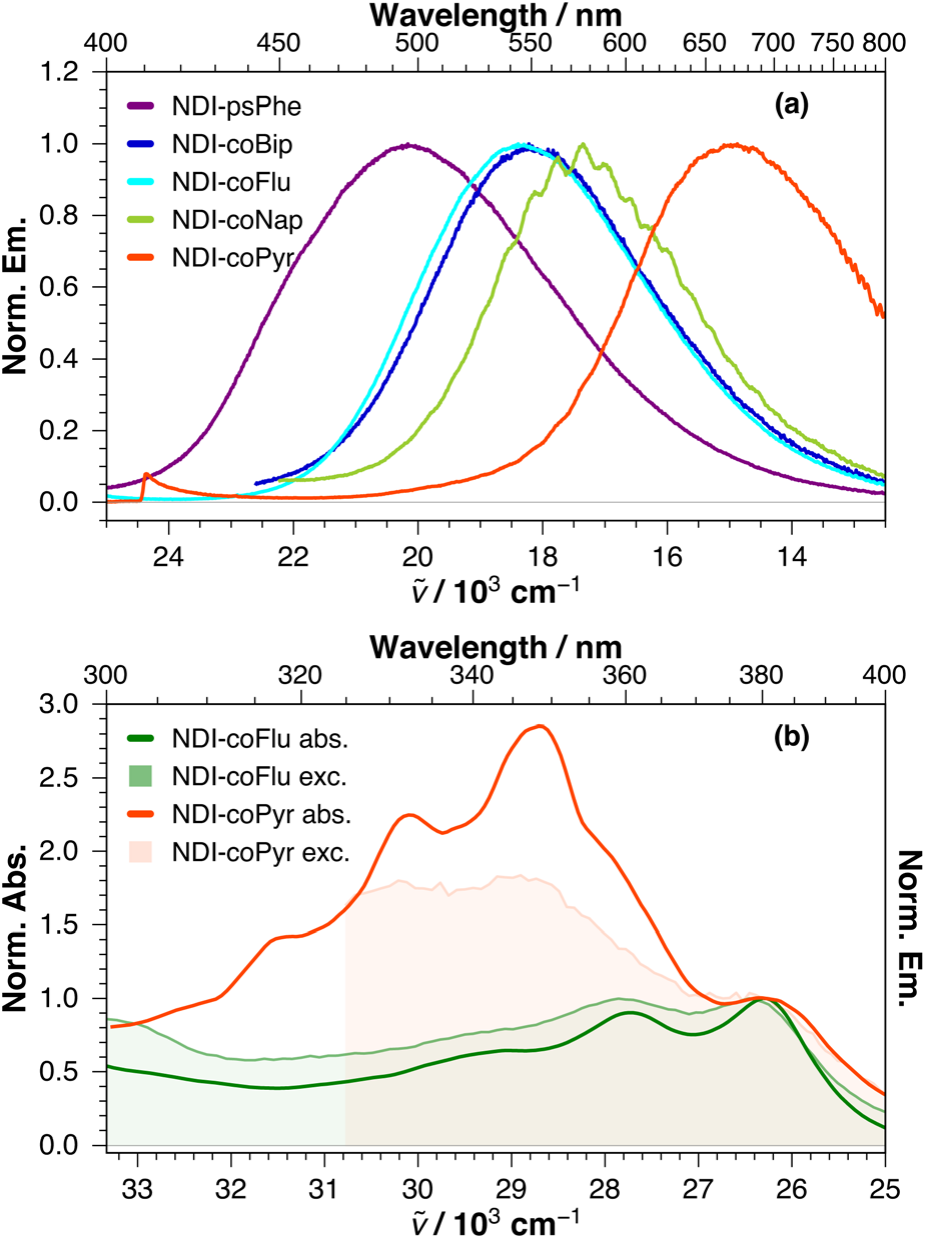
(a) Fluorescence spectra of (co)polymer films and (b) comparison of the fluorescence excitation and absorption spectra of NDI-coFlu and NDI-coPyr films.

The stationary electronic absorption spectra of the (co)polymers in DMF solutions and as films exhibit the characteristic structured S_1_ ← S_0_ band of the NDI acceptor with a maximum at around 380 nm (Fig. 2 and S1).^23^ For NDI-coFlu, NDI-coNap, and NDI-coBip, the absorption below 300 nm can be attributed to the secondary donor. The absorption spectrum of NDI-coPyr displays a broad band between 300 and 420 nm due to the overlapping contributions of both the NDI and pyrene chromophores (Fig. 3b and S1). The position and shape of the NDI band do not exhibit any significant dependence on the nature of D_1_ or D_2_. However, a marked broadening of this band is observed when going from the solutions to the films, with the onset of the S_1_ ← S_0_ band extending to 400 nm (Fig. 2). Given the nature of these molecules, this effect might arise from a CT band of D–A complexes present in the films. However, the absorption band maximum of such complexes is normally located at lower energy than those of the D–A constituents and shifts to lower energies as the D–A strength increases,^19,20,24^ contrary to what is observed here. On the other hand, similar band broadening was reported with NDI-based D–A and D–A–D molecules with one or two phenyl donors linked to the imide N atoms.^25^ This effect was shown to arise from the strong shortening of the excited-state lifetime of the NDI acceptor caused by an ultrafast ET occurring in a few tens of fs, a timescale comparable to that of electronic dephasing.^26–28^

The (co)polymers in solution are weakly emissive, with the exception of NDI-coPyr.^17^ The fluorescence spectrum of NDI-coPyr solutions exhibits the typical features of the pyrene monomer as well as a broad shoulder at around 480 nm due to the excimer (Fig. S1).^29^ As discussed in detail previously, the (co)polymer films are all emissive with a broad and structure-less band typical of CT fluorescence (Fig. 3a and S1).^17^ The band maximum correlates with the donating strength of the donors (D_1_ or D_2_), as expected for exciplex-like emission.^19,30^ The absence of such a dependence on the donor strength in the absorption spectrum suggests that band broadening is most probably due to the occurrence of ultrafast ET in the films rather than an overlapping CT absorption band.

This hypothesis is supported by the fluorescence excitation spectra of the films, which exhibit a significant band broadening compared to the absorption spectra (Fig. 3). Moreover, the excitation spectra do not present any feature that could be assigned to the CT band of D–A complexes. This difference between the excitation and absorption spectra implies that emission arises from a subset of molecules characterised by a broadened absorption band. Most probably, this sub-ensemble corresponds to highly coupled D–A pairs which undergo ultrafast ET. Such coupling implies a significant overlap of the molecular orbitals of the constituents, which results in a non-negligible transition dipole for CT emission. However, this dipole is probably too small to lead to a significant CT absorption band.

### 2.2 Transient absorption spectroscopy

Ultrafast transient electronic absorption (TA) measurements of (co)polymer solutions and films were first performed upon local excitation of the NDI acceptor at 380 nm. The slower dynamics in the films were investigated using ns to µs TA upon 355 nm excitation.^31^ The case of NDI-coPyr, where the secondary Pyr donor is also excited at 355 nm, will be discussed separately. The resulting TA data were analysed globally assuming a series of successive exponential steps (A → B → …) to obtain evolution-associated difference absorption spectra (EADS) and characteristic time constants. These EADS do not necessarily correspond to a given state or species but reflect the spectral changes and their timescales.^32,33^

**Fig. 4:**
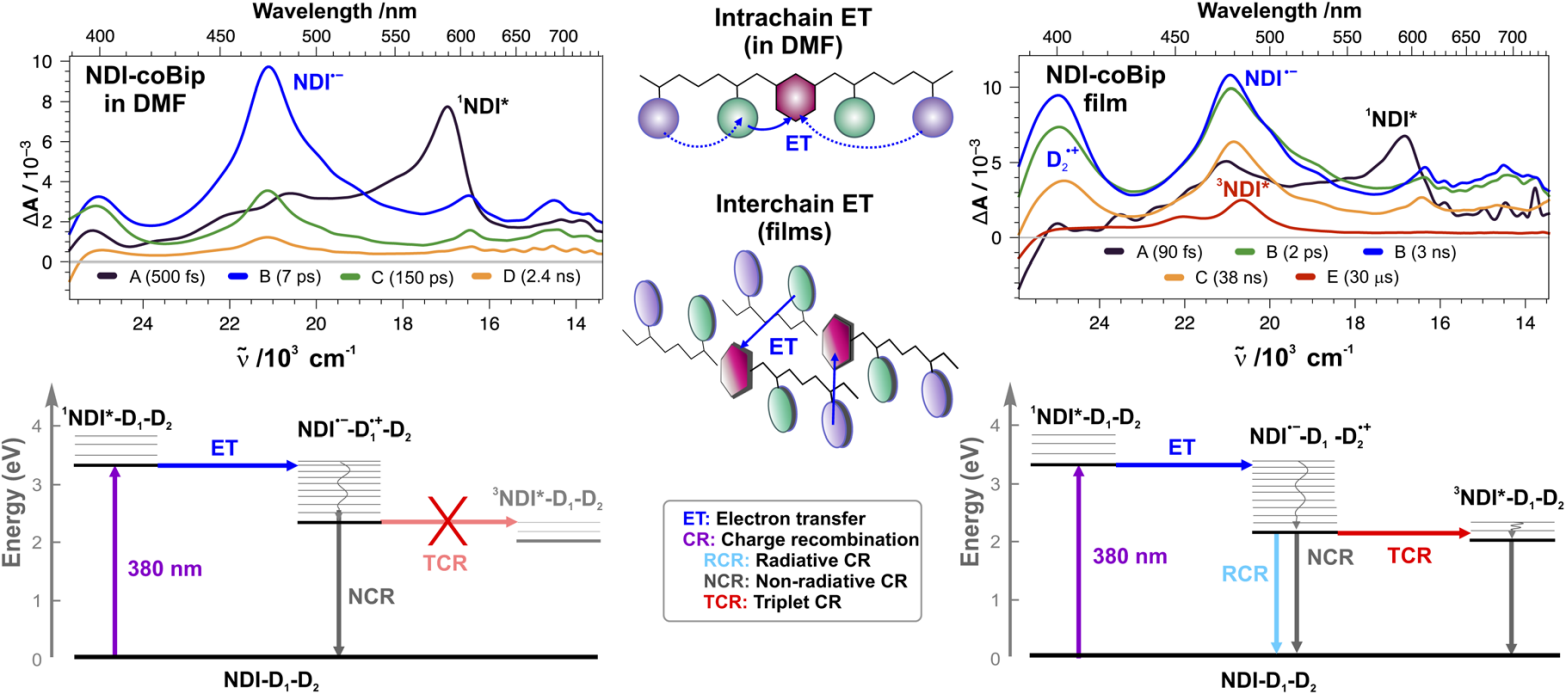
(Top) Evolution-associated difference absorption spectra (EADS) and time constants obtained from global analysis of the transient absorption data measured with NDI-coBip in DMF (left, 380 nm excitation) and as a film (right, 380 nm up to 2 ns, 355 nm after 2 ns) assuming a series of successive exponential steps (A → B → …). (Bottom) Energy-level diagrams illustrating the main processes occurring after photoexcitation of solutions (left) and films (right). (Middle) Schematic representation of interchain and intrachain electron transfer; the excited acceptor is depicted in pink, while the primary and secondary donors are in green and purple.

As illustrated in Fig. 4, S2 and S3, the early TA spectra in both solutions and films (EADS A) are dominated by a positive band peaking at around 585 nm, which can be attributed to S_*n*>1_ ← S_1_ excited state absorption (ESA) centred on the NDI acceptor.^34–36^ The onset of a negative signal due to ground state bleach (GSB) can also be observed below 390 nm. Finally, the structured S_1_ → S_0_ stimulated emission (SE) band of the NDI acceptor overlaps with the ESA background but is visible in the 400–440 nm region. These features of the locally excited (LE) acceptor, ^1^NDI*, decay in less than 1 ps, and a new spectrum appears concurrently. Independent of the (co)polymer, this spectrum (EADS B) exhibits an intense positive band centred at 470 nm with a weaker one at around 600 nm, both of which can be attributed to the radical anion NDI˙^−^.^35,37,38^ For copolymers with Bip and Flu as secondary donors, an additional positive band at around 400 nm is also visible. It is much more intense in the films than in solution and is the strongest with NDI-coBip (Fig. 4 and S2). This feature can be attributed to the radical cation of Bip˙^+^ and Flu˙^+^, in agreement with the literature.^39^ For the other (co)polymers, no clear spectral feature that could be attributed to the cation can be identified (Fig. S2). This can be explained by the weak absorbance of the radical cations of benzene and naphthalene relative to that of NDI˙^−^ within the spectral window of the experiment.^39–41^

Consequently, this first step (A → B) corresponds to an ultrafast ET to the locally excited NDI. The weak intensity of the cation band of D_2_ in solution suggests that the hole is mostly located on a nearby D_1_ donor. As D_2_ is generally situated further away from the acceptor along the chain compared to D_1_, proper folding of the polymer chain is most probably needed to achieve sufficient coupling with NDI for ET from D_2_ to be competitive with ET from D_1_. The sub-ps ET timescale in DMF is much shorter than that of the diffusional encounter between two polymer chains;^42,43^ therefore this ET is a through-space intrachain process (Fig. 4 middle).^44–47^ By contrast, the presence of the relatively intense cation band of D_2_ in the spectra measured a few hundreds of fs after excitation of NDI-coBip and NDI-coFlu films points to an intermolecular ET process from a D_2_ donor of one chain to a nearby ^1^NDI* of another polymer chain. Depending on the folding of the chains in the films, through-space intrachain ET could also be possible, but should not be dominant.

According to global analysis, ET (A → B step) in the copolymer films is faster than in the polymer films that lack D_2_ and is also faster than in all solutions, independent of the presence of a secondary donor (Fig. 4 and S3). No clear dependence on the nature of D_2_ of the dynamics is observed. The faster ET in copolymer films can be accounted for by a larger driving force, −Δ*G*_ET_, with D_2_ compared to that with the phenyl D_1_ donors (Table S2). ^1^NDI* is such a strong acceptor that ET with weak donors like benzene derivatives can occur on a sub-ps timescale,^25,48^ competing with ISC to the triplet state, ^3^NDI*, which is located about 1.3 eV below ^1^NDI*.^49^ The faster ET in films compared to DMF is more surprising considering that the weak polarity of the polymer medium (*ε* = 3 for polystyrene) compared to DMF (*ε* = 36.7) should result in a significantly smaller Δ*G*_ET_ in the film and, thus, to a slower ET.^50^ The faster ET in the films can be accounted for by a stronger electronic coupling favoured by the short D–A distance. As shown previously, intermolecular ET quenching between strongly coupled reactants can be as fast in non-polar as in highly-polar solvents.^51–54^

After the initial ET step (A → B), the dynamics in DMF and in films differ considerably. In DMF solutions, all spectral features decay entirely within a few ns. This decay occurs on several timescales with 5–10 ps, 100–200 ps and 1–2 ns components and can be attributed to charge recombination (CR) back to the neutral ground state. These multiple timescales point to a distribution of CR rate constants originating from a distribution of polymer conformations and a broad range of electronic coupling strengths. Additionally, given the large number of donors in the styrenic chains, the hole can, in principle, hop from its initial position to a nearby donor, thereby increasing the distance from the NDI˙^−^ and slowing down CR.

In contrast to DMF solutions, much slower CR is observed in the films. Typically, half of the amplitude of the ion bands decays on a few ns timescale, whereas the decay of the remainder is about ten times as slow (Fig. 4, S2 and S3). Furthermore, the decay of the ion bands is accompanied by the concurrent rise of a structured band with a maximum at around 480 nm (EADS E), which can be assigned to the triplet state of the NDI acceptor.^36,49^ This band and the residual GSB decay entirely in a few tens of µs.

Like in DMF, CR in the films occurs on multiple timescales, again most probably due to a distribution of electronic coupling strengths. The pronounced slowing down of CR relative to DMF can be accounted for by the polarity of the environment, which is strongly reduced when going from DMF to the films. Because of the difference in solvation energy, the CT state is at higher energy in the films than in DMF. This, in turn, leads to a correspondingly larger CR driving force, −Δ*G*_CR_, in the films, and, thus, to a significantly slower CR, as expected from Marcus theory for ET in the inverted region.^55–57^

The CR timescales are qualitatively similar to those reported previously for the CT fluorescence of the films,^17^ with no clear dependence on D_1_ and D_2_. On one hand, the ultrafast decay of the ^1^NDI* ESA band indicates that the entire S_1_ state population undergoes ET. On the other hand, the difference between the absorption and fluorescence excitation spectra implies that only a sub-ensemble of the CT state population is fluorescent. These observations are also consistent with a distribution of D–A conformations and coupling strengths. The most coupled D–A pairs undergo ultrafast ET to an emissive CT state, and thus have a broadened absorption band. This emissive state can be viewed as a highly coupled ion pair, with overlapping molecular orbitals.^22,51^ This allows for a non-negligible transition dipole moment for CT emission, like in exciplexes. For the remaining pairs, which are less coupled, ET is slower and results in a non-emissive CT state. The latter corresponds to a less coupled ion pair, with little overlap of the MOs and, thus, negligible emission dipole moment.

The higher energy and, consequently, longer lifetime of the CT state in the films compared to DMF can also account for the presence of ^3^NDI* in the late TA spectra. This triplet state is located 2.05 eV above the ground state,^49^ and is probably well below the CT state in the films, making CR to ^3^NDI* energetically feasible, with small to moderate driving force, *i.e.*, far from the inverted region (Fig. 4 bottom). Therefore, from a pure energetic point of view, CR to the triplet state should be much faster than the highly exergonic recombination to the ground state.

In principle, triplet recombination is forbidden because of the change in spin multiplicity. However, several mechanisms can enable this process: the heavy-atom effect,^58–60^ hyperfine coupling,^61,62^ and spin–orbit charge-transfer (SOCT) ISC.^63–66^ The first mechanism can be safely excluded because of the absence of heavy atoms near the donors and the acceptor. The hyperfine coupling mechanism requires the electron and hole to be sufficiently far apart for the singlet and triplet CT states to be degenerate. The exciplex-like nature of the CT state in the films results from a strong coupling of the ions and, therefore, points to non-degenerate singlet and triplet CT states. Consequently, the hyperfine coupling mechanism should not be operative here. SOCT ISC involves the motion of an electron between two orthogonal orbitals.^63–66^ The resulting change in orbital angular momentum can compensate for a change in spin. A non-coplanar mutual orientation of the molecular planes of the donor radical cation and NDI˙^−^ is quite probable in the films and should, thus, enable triplet CR *via* this mechanism. Given that triplet CR occurs on the tens of ns timescale in the films, its absence in DMF can also be accounted for by the much faster singlet CR, independent of whether the CT state in DMF is above or below ^3^NDI*.

The efficiency of the triplet recombination could not be determined precisely because of the strong spectral overlap of the different transients and the non-exponential dynamics. Nevertheless, triplet yields of the order of 20% could be estimated (Table S3). Given that the fluorescence quantum yield of these (co)polymers ranges from 3 to 10% (Table S3), one can conclude that non-radiative CR to the ground-state is the main decay pathway of the CT state. Slowing down these CR pathways could significantly enhance the emissive properties of these materials.

### 2.3 Photoselection of strongly coupled D–A pairs

The difference between the absorption and excitation spectra of the films suggests that the spectral broadening observed upon going from DMF solution to the film arise from the strongly coupled D–A pairs which undergo ultrafast ET. The existence of D–A pairs with different absorption spectra results in a inhomogeneous broadening of the NDI absorption band and this opens the possibility to photoselect distinct subpopulations.^67–69^ The above-described TA experiments were performed with pump pulses at 380 or 355 nm, exciting D–A pairs with a broad range of coupling strengths. However, excitation at the red edge of the NDI band should allow for the photoselection of the most coupled pairs, *i.e.*, those with the broadened spectrum. To test this hypothesis, we performed TA measurements upon 400 nm excitation, where no significant absorption is observed in DMF solutions (Fig. 2).

**Fig. 5:**
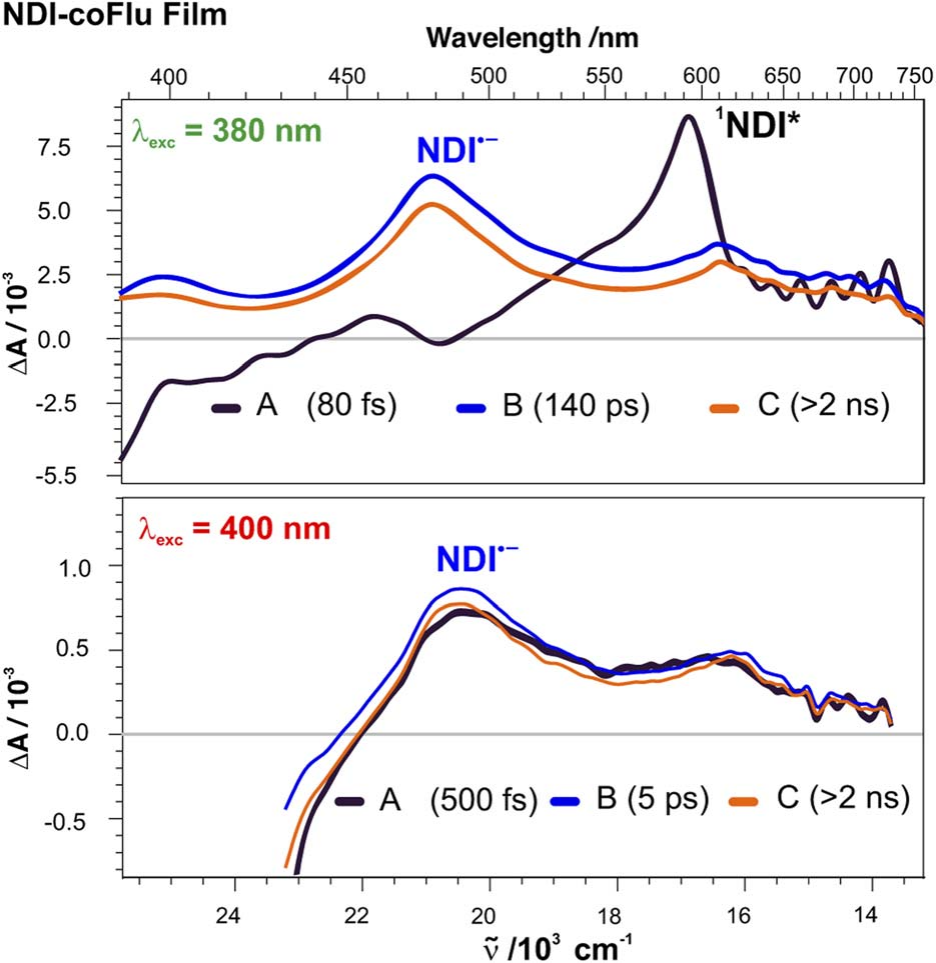
Evolution-associated difference absorption spectra and time constants obtained from a global analysis of the transient absorption data measured upon photoexcitation of a NDI-coFlu film at 380 nm (NDI band maximum, top) and 400 nm (red edge, bottom) assuming a series of three successive exponential steps (A → B → C →).

As illustrated in Fig. 5 with a NDI-coFlu film, the early TA spectra (EADS A) differ considerably from those measured upon 380 nm excitation, and do not exhibit the ESA band of ^1^NDI* at 585 nm. Instead, they resemble the spectra of the CT state recorded at later times upon 380 nm excitation and dominated by the NDI˙^−^ band at 490 nm. Very little dynamics are observed within 0–2 ns, the time window of the experiment, in agreement with relatively slow CR dynamics discussed above. Similar spectra were observed with the other copolymers with Nap and Bip as secondary donors (Fig. S4). In the case of the NDI-psD_1_ polymers, the absorbance of the films at 400 nm was too low to obtain good TA signals.

These data are consistent with photoinduced ET occurring in a few tens of fs as expected for strongly coupled D–A pairs.^25,48^ This is too fast to be resolved here with the 100–200 fs response function of the TA setup, and therefore the 585 nm band of ^1^NDI* is not visible. Of course, these strongly coupled pairs also absorb at 380 nm, but their relative population is too small to contribute significantly to the TA signal. This is why no clear spectral feature of the CT state can be identified in the early spectra. Further TA measurements upon 355 nm excitation, *i.e.* on the blue side of the NDI band, were performed to find out whether different distributions of D–A pairs are photoselected. As illustrated in Fig. S4 and S5, the early TA spectra resemble those recorded upon 380 nm excitation, with the spectral features of ^1^NDI*. However, the relative amplitude of the ^1^NDI* band is weaker and the background signal in the 450–550 nm region could possibly be due to the presence of the NDI˙^−^ band. Consequently, although the populations excited at 355 nm are not very different than those excited at 380 nm, a higher contribution of strongly coupled pairs cannot be excluded.

### 2.4 Pyrene copolymer: exciting both the acceptor and the secondary donor

Because of the relatively low energy of the S_1_ state of Pyr, we can use NDI-coPyr to investigate the excited-state dynamics upon excitation of D_2_. According to the electronic absorption spectrum of NDI-coPyr, both NDI and Pyr should be photoexcited at 355 nm, while light at 380 nm should mostly interact with NDI (Fig. 2).

**Fig. 6:**
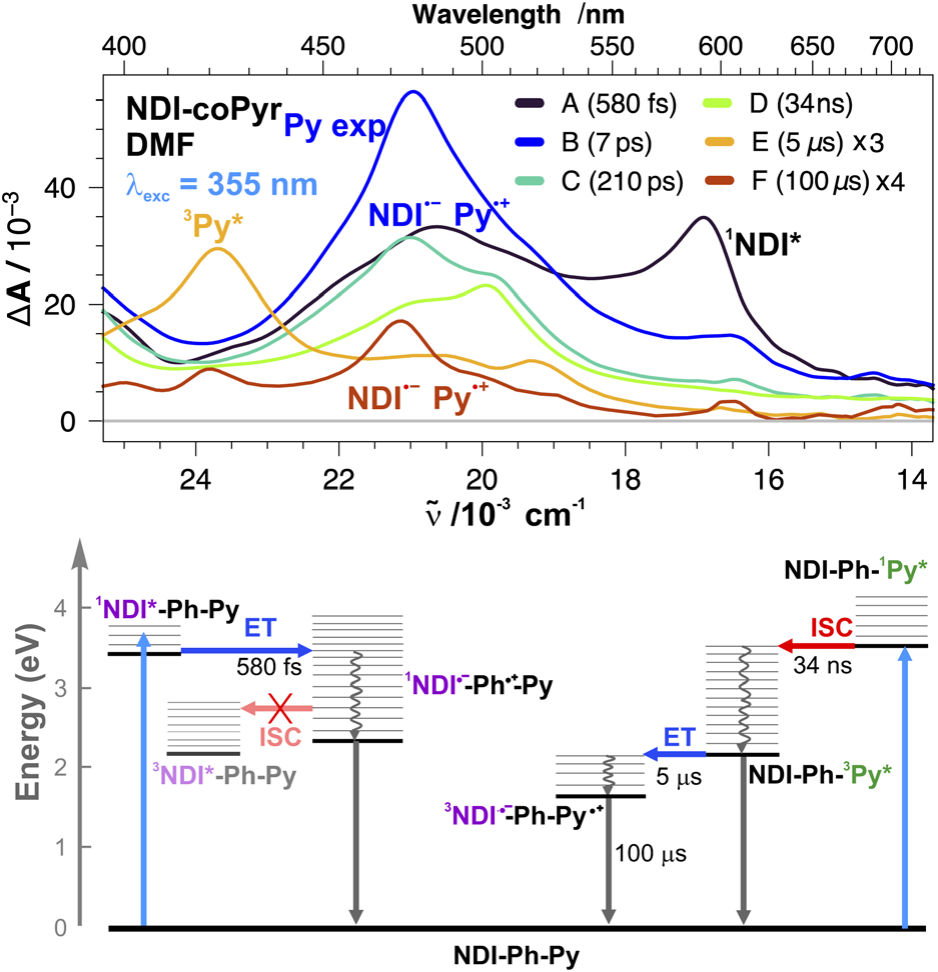
(Top) Evolution-associated difference absorption spectra and time constants obtained from a global analysis of the transient absorption data measured upon 355 nm excitation of NDI-coPyr in DMF solution, assuming a series of six successive exponential steps (A → B → … → F →). (Bottom) Energy-level diagrams with the most relevant processes.

The TA spectra recorded upon 380 nm excitation of NDI-coPyr in DMF indicate that the excited-state dynamics are very similar to those of the other (co)polymers in DMF, namely, ultrafast ET followed by complete CR within a few ns (Fig. S6). Significantly more complex dynamics are observed upon 355 nm excitation and a series of not less than six consecutive exponential steps had to be used to reproduce the TA data (Fig. 6). The early spectra (EADS A) show the ^1^NDI* ESA band at 585 nm as well as a band peaking at ∼490 nm that is broader and more intense than that when excited at 380 nm (Fig. S6). This feature is most probably due to multiple contributions, such as ^1^NDI*, NDI˙^−^ and ^1^Pyr*, known to absorb in the 450–510 nm range.^70,71^ This early spectrum evolves in <1 ps to a spectrum dominated by the NDI˙^−^ bands at 470 and 600 nm (EADS B). However, a shoulder at around 500 nm, most probably due to ^1^Pyr*, is also present. The NDI˙^−^ bands decay almost entirely during the next few hundreds of ps, whereas the intensity at around 500 nm does not change much. Consequently, the ^1^Pyr* 500 nm shoulder becomes a well-defined band (steps B → C → D). The latter transforms in about 30 ns to a band at around 420 nm (D → E), which is consistent with the triplet state of pyrene, ^3^Pyr*.^72^ The decay of this band in 5 µs (E → F) is accompanied by the reappearance of the NDI˙^−^ bands, which finally decay in about 100 µs.

These complicated dynamics can be rationalised by considering that they correspond to the superposition of two parallel photocycles, associated with the excitation of NDI and Pyr (Fig. 6). The dynamics following the excitation of NDI should be qualitatively the same as those observed with 380 nm pump pulses, with the ultrafast population of the CT state, with the hole most probably on a D_1_ donor, followed by CR within a few ns.

Photoexcitation of Pyr leads to the population of ^1^Pyr* with an ESA band at around 500 nm.^70,71^^1^Pyr* decays in a few tens of ns *via* fluorescence and internal conversion to the ground state as well as *via* ISC to ^3^Pyr*. This decay is faster than that reported for Pyr in solution,^73^ and could also involve excimer formation, in agreement with the presence of the Pyr excimer band in the fluorescence spectrum, as well as ET with NDI. Unfortunately, both the Pyr excimer and Pyr˙^+^ are also characterised by an absorption band at around 500 nm,^39,74,75^ and, therefore, their presence cannot be established with certainty. In any case, only ^3^Pyr* is significantly populated on the few hundreds of ns timescale. The transformation of the ^3^Pyr* band to the NDI˙^−^ band in 5 µs (E → F) can be interpreted as an ET from ^3^Pyr* to NDI. CR of the resulting triplet CT state to the ground state is spin forbidden and is thus much slower than CR of the singlet CT state populated after photoexcitation of NDI.

The relatively weak intensity of the late NDI˙^−^ band and its very slow increase point to a low efficiency of the ET from ^3^Pyr*. The driving force of this process, estimated to be around 0.25 eV (Table S1), is too large to explain a µs ET time constant. The inefficiency of this process can rather be explained by the large distance between the NDI acceptor and the Pyr secondary donors. Most probably, structural fluctuations of the polymer chains and/or hopping of the triplet energy are required to bring ^3^Pyr* and NDI to ET distances. Finally, the triplet CT state should be about 0.2 eV below ^3^NDI* and, therefore, spin-allowed CR to ^3^NDI* is not possible. Consequently, the triplet CT state can only recombine non-radiatively to the ground state.

**Fig. 7:**
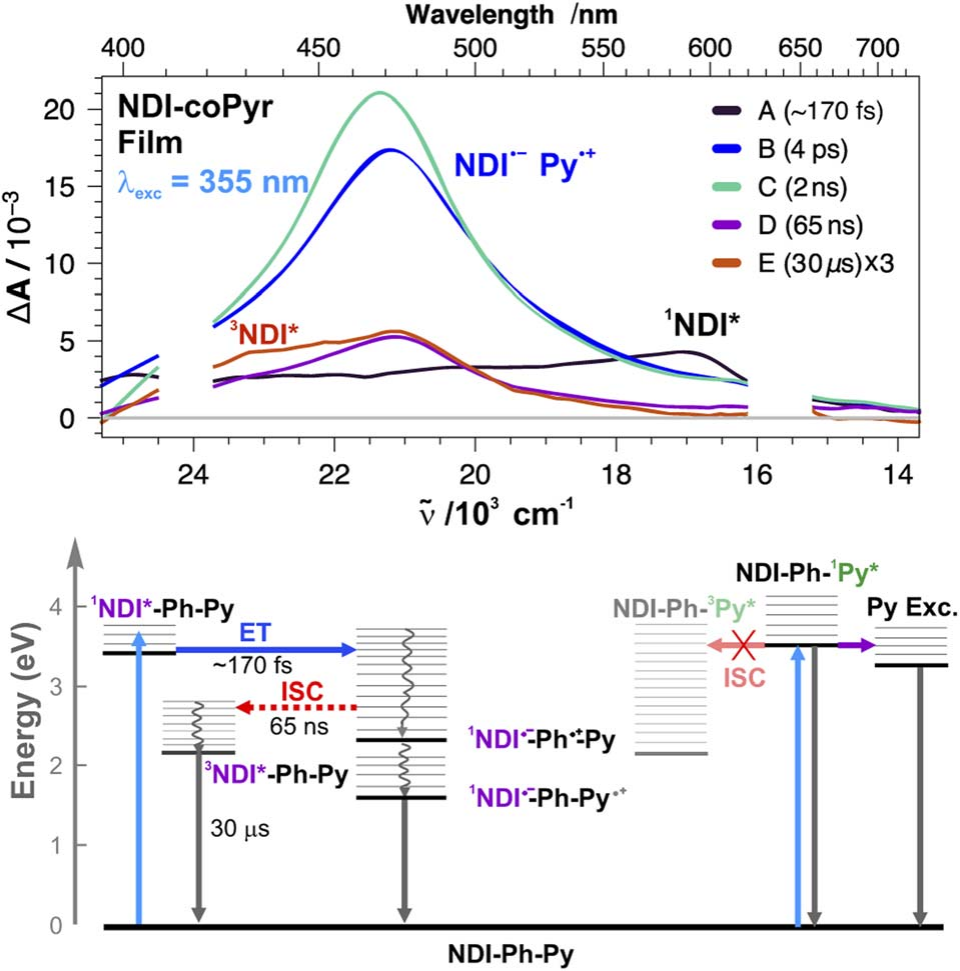
(Top) Evolution-associated difference absorption spectra and time constants obtained from a global analysis of the transient absorption data measured upon 355 nm excitation of a NDI-coPyr film, assuming a series of five successive exponential steps (A → B → … → E →). (Bottom) Energy-level diagrams with the most relevant processes.

Simpler dynamics are observed upon 355 nm excitation of NDI-coPyr films (Fig. 7). Apart from the 585 nm ESA band of ^1^NDI* visible during the first few hundreds of fs, the TA spectra are dominated by a band peaking at 470 nm, which is significantly broader than that upon 380 nm excitation, suggesting the contributions of Pyr transients, such as ^1^Pyr* and the excimer, overlapping with the NDI˙^−^ band. Excimer formation is consistent with the presence of the excimer band in the stationary fluorescence spectrum of the film. This broad TA band decays on multiple timescales to a weak residual spectrum, exhibiting the ^3^NDI* band and decaying on the 30 µs timescale. Given that various transients could contribute to this broad band, the interpretation of the TA dynamics is not obvious. Overall, the observed dynamics are not largely different from those measured with the other (co)polymer films. No evidence of ^3^Pyr* can be detected here. Most probably, direct excitation of Pyr results mainly in the formation of excimers, which decay radiatively and non-radiatively to the ground state. Such excimer formation in the films is probably favoured by the interchain interactions that are less probable in solution. Although ET from ^1^Pyr* cannot be ruled out, it should be noted that, given the composition of this copolymer, an excited Pyr subunit is statistically more likely to form an excimer with a nearby non-excited Pyr than to undergo ET with a less abundant NDI acceptor.

A major difference with the other (co)polymer films is the much smaller amplitude of the ^3^NDI* band. Apart from fact that direct Pyr excitation does not result in the population of the CT state, this smaller triplet yield could be due to the better electron donating ability of Pyr compared to the other donors. Because of this, CR to the ground state should be less in the inverted region and, thus, be faster, in agreement with the shorter fluorescence lifetime reported previously.^17^ Furthermore, triplet recombination to ^3^NDI* should become more weakly exergonic and, thus, slower.

These results reveal that direct excitation of the secondary donor is detrimental to the CT emission yield of these films. The highest efficiency is obtained on directly exciting the NDI acceptor. The secondary donors act like internal filters and, upon excitation, decay along pathways that do not lead to the population of the emissive CT state.

## 3 Conclusions

This work is the first detailed study of the photophysical properties of this new type of non-conjugated D–A polymer, providing unequivocal insights into the origin of their tunable fluorescence. Our results reveal that the excited-state dynamics depend strongly on whether these (co)polymers are diluted in solution or prepared as films, *i.e.*, whether interchain interactions are present. In both cases, ultrafast electron transfer to the initially photoexcited acceptor is taking place. We show that, in solution, this electron transfer is an intrachain process and occurs with a nearby styrenic donor. By contrast, electron transfer in films is an interchain process and can take place with the stronger secondary donors, which are located far away from the acceptor along the chain. We found that the charge-transfer emission of the films originated from strongly coupled donor–acceptor pairs for which ET is fast enough to broaden the absorption band of the acceptor. Photoselection of these highly coupled pairs upon red-edge excitation was demonstrated.

The charge-transfer state is strongly stabilised in polar solution and, thus, undergoes sub-ns charge recombination to the ground state. By contrast, it is at higher energy in the films and its recombination occurs on the tens of ns timescale. We found that a significant fraction of the charge-transfer state population recombines to the triplet state of the acceptor. This triplet recombination competes with the radiative recombination and is detrimental to the fluorescence quantum yield of these materials. This unwanted decay pathway can be significantly reduced when using a strong secondary donor. However, this approach automatically leads to low-energy emission. Another strategy would be to use an acceptor with a higher triplet energy.

Finally, we found that direct photoexcitation of the donor is prejudicial to the fluorescence quantum yield, because, as an excited donor has high probability to be situated far from an acceptor, it can decay without undergoing electron transfer.

In addition to elucidating the origin of the luminescence of these (co)polymers, our work contributes to a deeper understanding of the photodynamics of electron donor–acceptor polymers, which should be useful for further improvements as well as for the development of new photoactive polymeric materials with specific properties.

## Author contributions

E. Sucre-Rosales designed and performed the spectroscopic experiments, analysed the data and wrote the initial draft. S. Ye synthesised the compounds and prepared the polymer films under the supervision of Y. Bao. E. Vauthey supervised the spectroscopic study and wrote the final version with the help of all authors.

## Conflicts of interest

There are no conflicts to declare.

## Supplementary Material

SC-OLF-D5SC07237A-s001

## Data Availability

All data can be downloaded from https://doi.org/10.26037/yareta:vhpfezelunby7k52hflhmezele. Supplementary information (SI) is available. See DOI: https://doi.org/10.1039/d5sc07237a.
