# Supplementary material for: Photoinduced charge-transfer dynamics in fluorescent electron donor–acceptor polymers
Source: Chem Sci. 2025 Nov 25;17(4):2119–27. doi: 10.1039/d5sc07237a (PMC12671088; doi:10.1039/d5sc07237a)
Supplement: SC-017-D5SC07237A-s001 [file SC-017-D5SC07237A-s001.pdf]

Supplementary Information (SI) for Chemical Science.
This journal is © The Royal Society of Chemistry 2025

Photoinduced Charge-Transfer Dynamics in Fluorescent Electron Donor-Acceptor Polymers

Estefanía Sucre-Rosales,[a] Suiying Ye,[b], Yinyin Bao,*[b,c] and Eric Vauthey*[a]

[a]Department of Physical Chemistry, University of Geneva, 30 Quai Ernest-Ansermet, CH-1211 Geneva 4, Switzerland. E-mail: eric.vauthey@unige.ch

[b] Institute of Pharmaceutical Sciences, Department of Chemistry and Applied Biosciences, ETH Zurich, Vladimir-Prelog-Weg 3, Zurich, 8093 Switzerland.

[c]Department of Chemistry, Faculty of Science, University of Helsinki, A. I. Virtasen aukio 1, 00014 Helsinki, Finland. E-mail: yinyin.bao@helsinki.fi

Contents

S1 Experimental Methods 3
S1.1 Samples and solvents . . . 3
S1.2 Stationary Electronic Spectroscopy . . . 3
S1.3 Transient Electronic Absorption Spectroscopy (TA) . . . 3
S1.3.1 fs-ps TA . . . 3
S1.3.2 ps-μs TA . . . 4
S1.3.3 Additional details . . . 4
S1.4 Energetics of the photoinduced electron transfer . . . 5
S1.5 Fluorescence and triplet quantum yields . . . 5

S2 Additional Figures 6
S2.1 Stationary electronic absorption and fluorescence emission spectra . . . 6
S2.2 Transient absorption spectroscopy . . . 7
S2.2.1 Liquid solutions vs. films . . . 7
S2.2.2 Transient absorption at different excitation wavelengths . . . 9

List of Figures

S1 Stationary absorption (continuous lines) and fluorescence emission spectra (dotted lines) of polymer solutions in DMF (upper) and of spin-coated (SC) films (bottom). The fluorescence spectra in DMF and films were measured upon 355 and 380 nm excitation, respectively. 6
S2 Transient absorption spectra measured upon excitation of the (co)polymers in DMF (top) and films (middle and bottom) upon 380 nm (0 to 2 ns) and 355 nm excitation (films only, from ns to 500 μs). 7
S3 Evolution-associated difference absorption spectra and time constants obtained from a sequential global analysis with four to five steps of the TA data in DMF (upper) and in spin-coated films (bottom) after 380 nm excitation (from fs to 2 ns) and 355 nm excitation (films only, from ns to 100 μs). The two sets of data were merged before performing the global analysis. 8
S4 Transient absorption spectra measured with the (co)polymer films after 355, 380 and 400 nm excitation. 9
S5 Evolution-associated difference absorption spectra and time constants obtained from a sequential global analysis with 2 (A $\rightarrow$ B $\rightarrow$), or 3 (A $\rightarrow$ B $\rightarrow$ C $\rightarrow$) steps of of the TA data measured with the (co)polymer films at various excitation wavelengths. 10
S6 Transient absorption spectra measured after 355 nm excitation of the (co)polymers in DMF solutions. 11
S7 Evolution-associated difference absorption spectra and time constants obtained from a sequential global analysis with 3 (A $\rightarrow$ B $\rightarrow$ C $\rightarrow$), 4 (A $\rightarrow$ B $\rightarrow$ C $\rightarrow$ D $\rightarrow$) or 6 (A $\rightarrow$ B $\rightarrow$ C $\rightarrow$ D $\rightarrow$ E $\rightarrow$ F $\rightarrow$) steps of the TA data measured with the (co)polymer in DMF solution at various excitation wavelengths. 12

S1 Experimental Methods

S1.1 Samples and solvents

The (co)polymers were synthesized and purified according to the protocol described in ref. 1,2. Briefly, atom transfer radical polymerizations were carried out using NDI-diBr (1 equiv) as the initiator, copper(I) bromide (1.5 equiv) as the catalyst, and 4,4'-dinoyl-2,2'-dipyridyl (1.5 equiv) as the ligand. Styrene (St) and 3-methylstyrene (MeSt) were used for homopolymerization, while the polycyclic aromatic monomers (PAMs), including 4-vinylbiphenyl, 9-fluorenyl methacrylate, 1-vinylnaphthalene, and 1-pyrenemethyl methacrylate, were used for statistical copolymerization with styrene, which also served as the reaction solvent. All solids were first loaded in a dry Schlenk flask, which was evacuated and refilled with argon for several times to remove oxygen. Under argon flow, the desired amounts of degassed monomers were added to the Schlenk flask. The reaction was carried out in an oil bath at 80 °C overnight. Detailed characterizations of the polymers can be found in ref. 1,2 and are also listed in Table S1.

Table S1 Characteristics of the (co)polymers

Sample	DP_{St} [a]	DP_{MeSt} [a]	DP_{PAM} [a]	PAM[b] loading	$M_{n,NMR}$ [c] (g mol^{-1})	$M_{n,GPC}$ [d] (g mol^{-1})	Đ[d]
**NDI-psPhe**	18	-	-		2520	2710	1.12
**NDI-psTol**	-	20	-		3020	2240	1.06
**NDI-coBip**	53	-	5.7	9.80%	6510	6480	1.13
**NDI-coFlu**	28	-	2.2	7.40%	3420	3790	1.15
**NDI-coNap**	23	-	1.8	7.30%	2620	3120	1.34
**NDI-coPyr**	24	-	1.9	7.20%	3090	2560	1.15

[a)]Degree of polymerisation (DP) determined by ^{1}H-NMR and estimated from the corresponding ratio of areas under the peaks at 6.45-7.29 ppm (phenyl protons in polystyrene), at characteristic peaks of PAMs, and at 8.75 ppm (aromatic protons in NDI scaffold). [b)]PAM loading corresponding to the ratio between St and PAM based on the estimated DP from NMR. [c)] Average molar mass calculated as: $M_{n,NMR} = MW_{NDI-diBr} + DP_{St,NMR} \times MW_{styrene} + DP_{PAM,NMR} \times MW_{PAM}$. [d)]$M_{n,GPC}$ and polydispersity index (Đ) determined by gel permeation chromatography (GPC), calibrated with poly(methyl methacrylate) standards.

Polymer thin films were prepared by spin-coating. Typically, quartz substrates with a size of 20 × 20 mm were brush-cleaned, sonicated in acetone and isopropanol, followed by drying on a hot plate at 120 °C for 20 min. Polymer in toluene solutions (70 mg/mL) were spin-casted at a spin rate of 1500 rpm for 30 s at the first step and 2500 rpm for 20 s at the second step, yielding thin films with a thickness ranging from 50 to 150 nm.

N,N'-dimethylformamide (DMF) was of spectroscopic grade, and was used as received. The polymer solutions were prepared with an absorbance of 0.4 at 380 nm.

S1.2 Stationary Electronic Spectroscopy

All samples were measured in 1 cm quartz cuvettes. The stationary electronic absorption spectra were measured using a Cary 50 spectrometer, while the stationary emission spectra were recorded on a Horiba FluoroMax-4 spectrofluorometer and corrected using a set of secondary emissive standards.

S1.3 Transient Electronic Absorption Spectroscopy (TA)

S1.3.1 fs-ps TA

The TA setup was described in detail in ref. **?** . Briefly, excitation was performed at 355 and 380 nm by doubling the output of a TOPAS-Prime combined with a NirUVis frequency mixer (both from Light Conversion), itself seeded by the output of a 5 kHz Ti:Sapphire amplified system (Spectra Physics, Solstice Ace). The excitation at 400 nm was achieved by doubling the fundamental output of the above-mentioned amplifier.

S1.3.2 ps-μs TA

The ps-μ pump-probe setup is described in detail in ref. 3. In brief, excitation was achieved using the output of a passively Q-switched, frequency tripled Nd:YAG laser (Teem Photonics, Powerchip NanoUV) producing pulses at 355 nm with a 500 Hz repetition rate, approximately 20 μJ energy, and 300 ps duration.

S1.3.3 Additional details

All samples solution were kept under nitrogen flow during the entire duration of the measurement (around 1.5-2 h). The films were moved in Lissajous figures during the measurement. The transient absorption signal was checked prior to measurement to scale linearly with the pump irradiance on the sample. The polarization of the pump pulses was set to magic angle relative to the white-light pulses. For the data treatment, the pixel-to-wavelength conversion was done using a standard Holmium oxide filter, which shows narrow bands in the UV-Vis spectral region. For all TA measurements, the global analysis was performed using a home-written script in Matlab®[4].

S1.4 Energetics of the photoinduced electron transfer

The driving force for photoinduced electron transfer, ΔG_{ET}, can be estimated using the Weller equation:

$$\Delta G_{\mathrm{ET}} = e[E_0(\mathrm{D}^+/\mathrm{D}) - E_0(\mathrm{A}/\mathrm{A}^-)] - E_{0,0} \quad \text{(S1)}$$

where $E_{0,0}$ is the energy of the excited-state, and $E_0(\mathrm{D}^+/\mathrm{D})$ and $E_0(\mathrm{A}/\mathrm{A}^-)$ are the oxidation and reduction potentials of the donor and acceptor, respectively.

Table S2 Excited-state energies, redox potentials and ET driving forces

	$E_{0,0}$ / eV	E_0 / V vs. SCE	-ΔG_{ET}(D-^{1}NDI*) / eV	-ΔG_{ET}(^{3}D*-NDI) / eV
^{1}NDI	3.2			
^{3}NDI	2.05 ^{a}			
^{1}Py	3.58			
^{3}Py	2.09 ^{b}			
NDI/NDI$^{\bullet-}$		-0.55 ^{c}		
Phe/Phe$^{\bullet+}$		2.48 ^{d}	0.17	
Tol/Tol$^{\bullet+}$		2.26 ^{d}	0.39	
Bip/Bip$^{\bullet+}$		1.95 ^{d}	0.70	
Flu/Flu$^{\bullet+}$		1.80 ^{e}	0.85	
Nap/Nap$^{\bullet+}$		1.75 ^{f}	0.90	
Pyr/Pyr$^{\bullet+}$		1.29 ^{f}	1.36	0.25

$^{a)}$ From ref. 5; $^{b)}$ from ref. 6; $^{c)}$ from ref. 7; $^{d)}$ from ref. 8; $^{e)}$ from ref. 9; $^{f)}$ from ref. 10.

S1.5 Fluorescence and triplet quantum yields

The fluorescence quantum yields (ϕ_f) are taken from ref. 1,2. The triplet quantum yields ($\phi_{^3NDI}$) were estimated using the intensity ratio of the EADS of ^{3}NDI (E→) and of the first EADS of NDI$^{\bullet-}$ (B→C), and compared to the triplet yield of NDI in ACN (70 %) reported in ref. 11.

Table S3 Fluorescence and triplet quantum yields

Polymer	ϕ_{fl}	ϕ_t
NDI-psPhe	0.05	0.23
NDI-psTol	0.05	0.22
NDI-coBip	0.08	0.24
NDI-coFlu	0.11	0.16
NDI-coNap	0.5	-^{a}
NDI-coPyr	0.03	-^{b}

$^{a)}$ Not calculated due to the presence of ^{3}NDI spectral signatures in the EADS corresponding to step (D→E). $^{b)}$ Not calculated due to the overlapping excimer signal.

S2 Additional Figures

S2.1 Stationary electronic absorption and fluorescence emission spectra

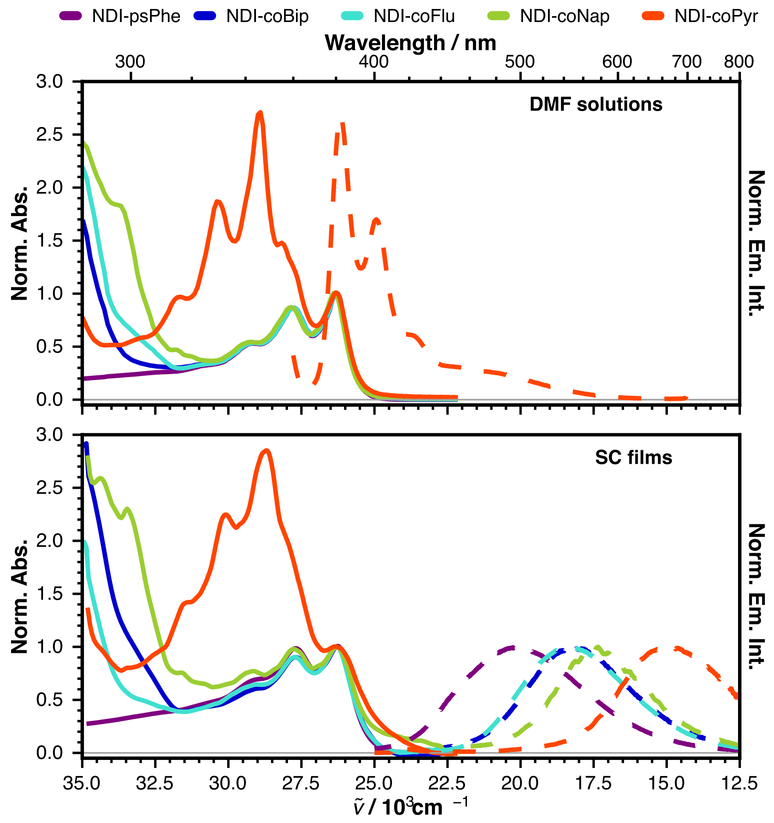

Figure S1 Stationary absorption (continuous lines) and fluorescence emission spectra (dotted lines) of polymer solutions in DMF (upper) and of spin-coated (SC) films (bottom). The fluorescence spectra in DMF and films were measured upon 355 and 380 nm excitation, respectively.

S2.2 Transient absorption spectroscopy

S2.2.1 Liquid solutions vs. films

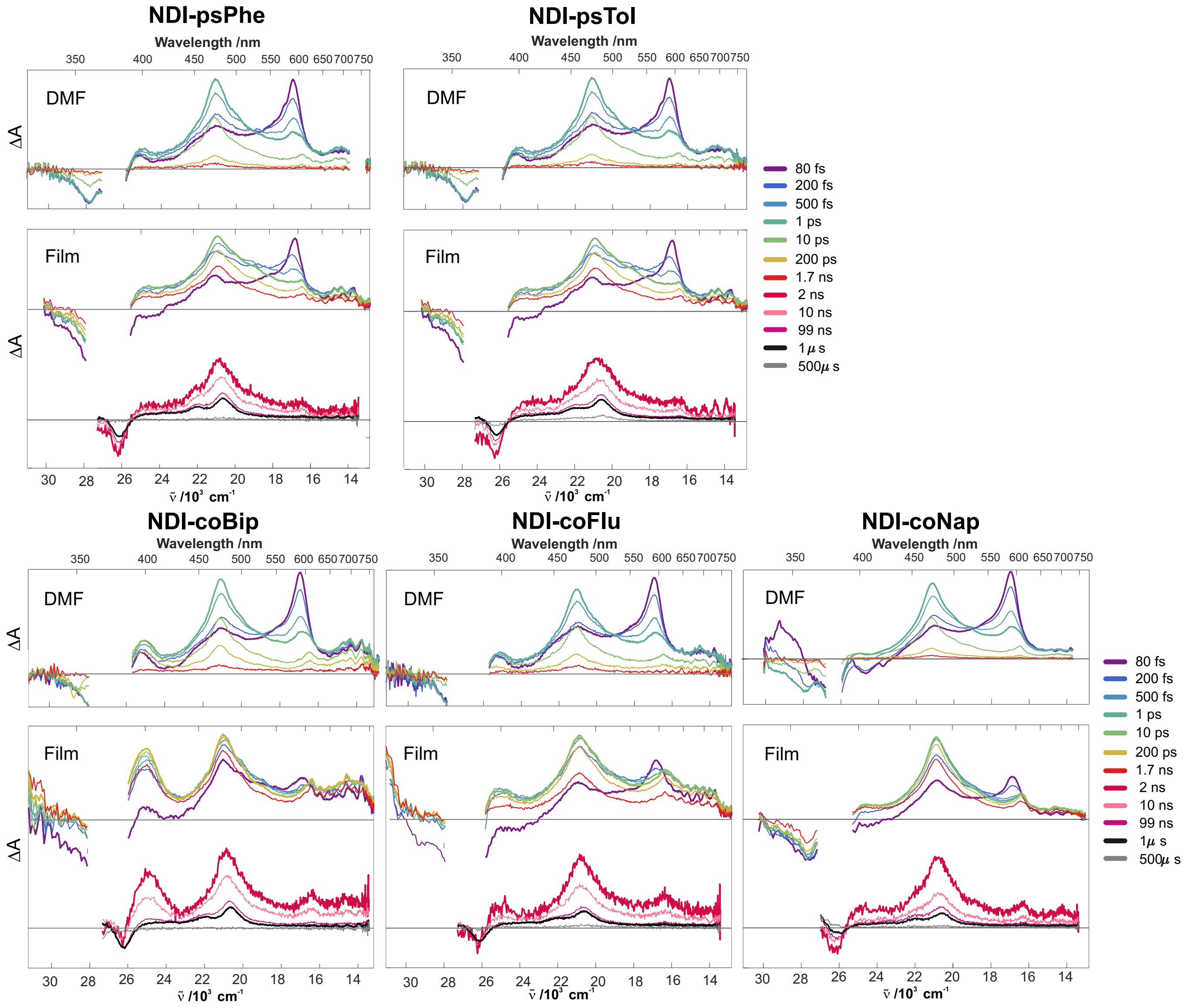

Figure S2 Transient absorption spectra measured upon excitation of the (co)polymers in DMF (top) and films (middle and bottom) upon 380 nm (0 to 2 ns) and 355 nm excitation (films only, from ns to 500 μs).

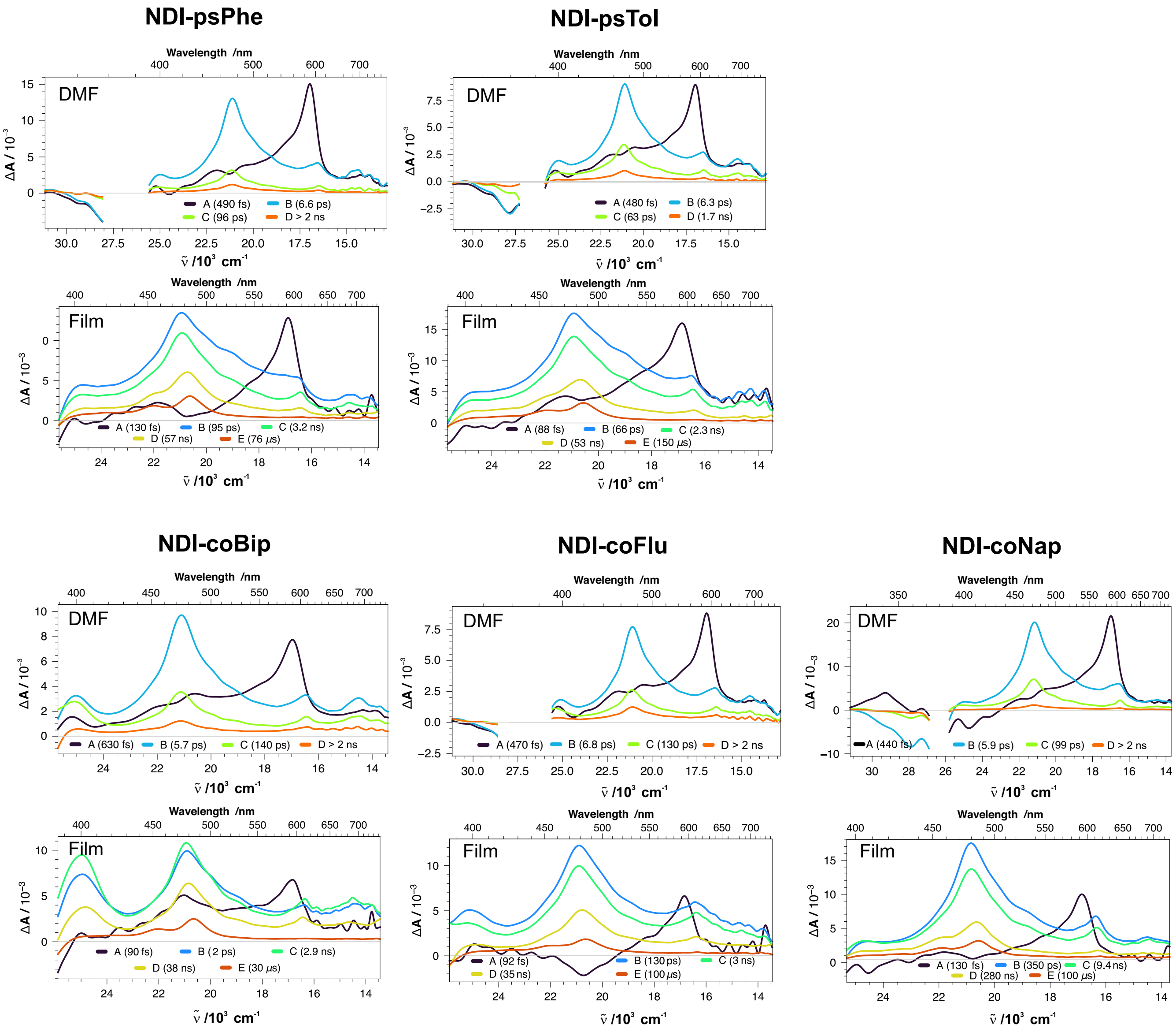

Figure S3 Evolution-associated difference absorption spectra and time constants obtained from a sequential global analysis with four to five steps of the TA data in DMF (upper) and in spin-coated films (bottom) after 380 nm excitation (from fs to 2 ns) and 355 nm excitation (films only, from ns to 100 μs). The two sets of data were merged before performing the global analysis.

S2.2.2 Transient absorption at different excitation wavelengths

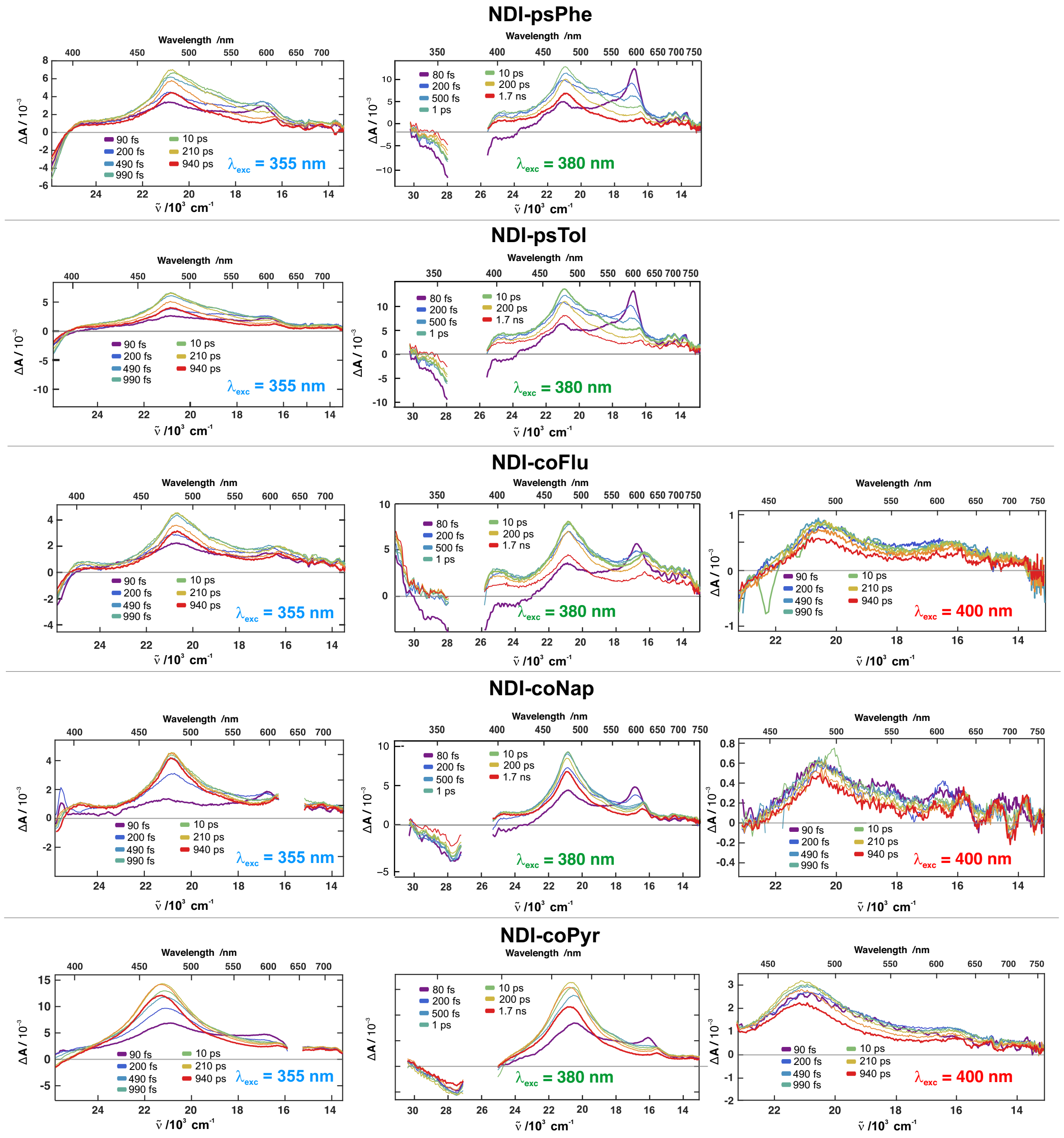

Figure S4 Transient absorption spectra measured with the (co)polymer films after 355, 380 and 400 nm excitation.

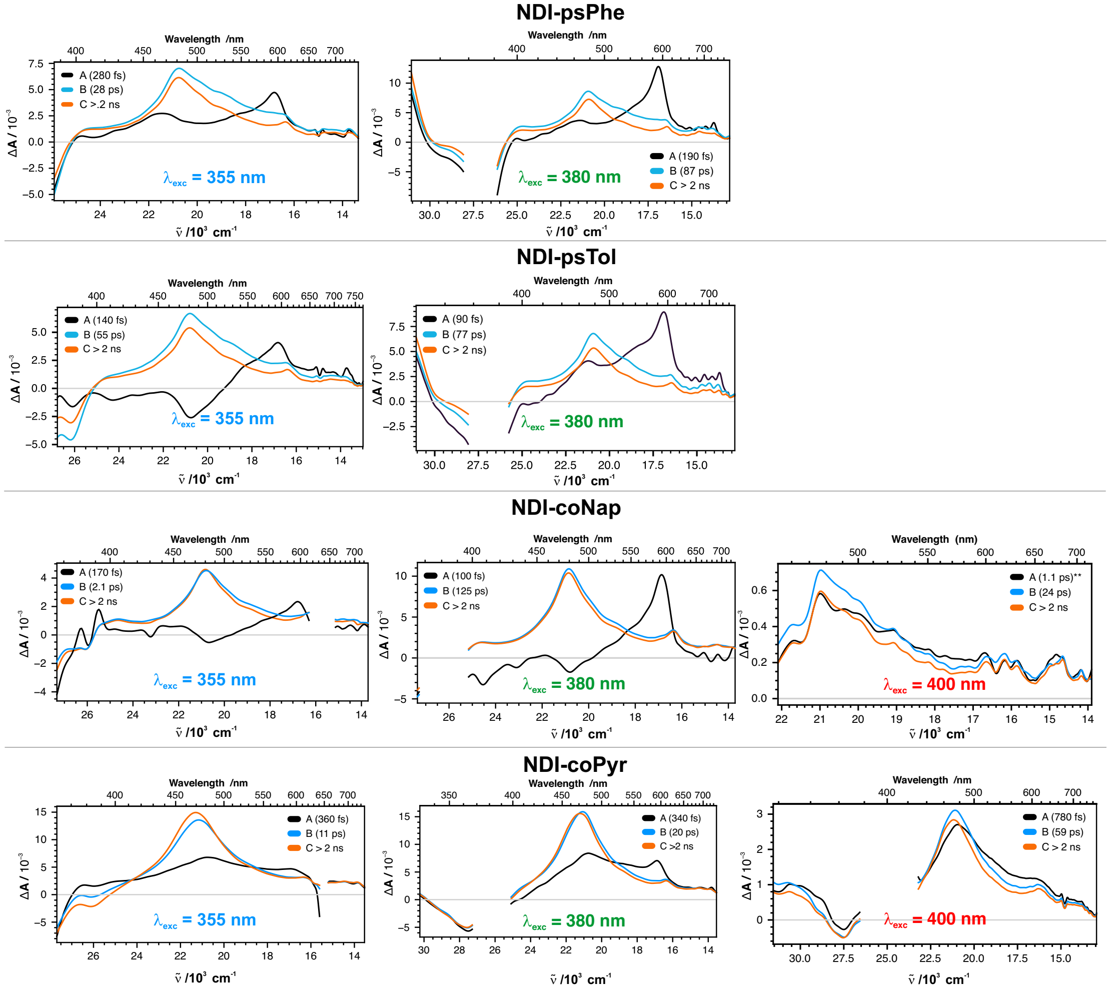

Figure S5 Evolution-associated difference absorption spectra and time constants obtained from a sequential global analysis with 2 ($A \rightarrow B \rightarrow$), or 3 ($A \rightarrow B \rightarrow C \rightarrow$) steps of of the TA data measured with the (co)polymer films at various excitation wavelengths.

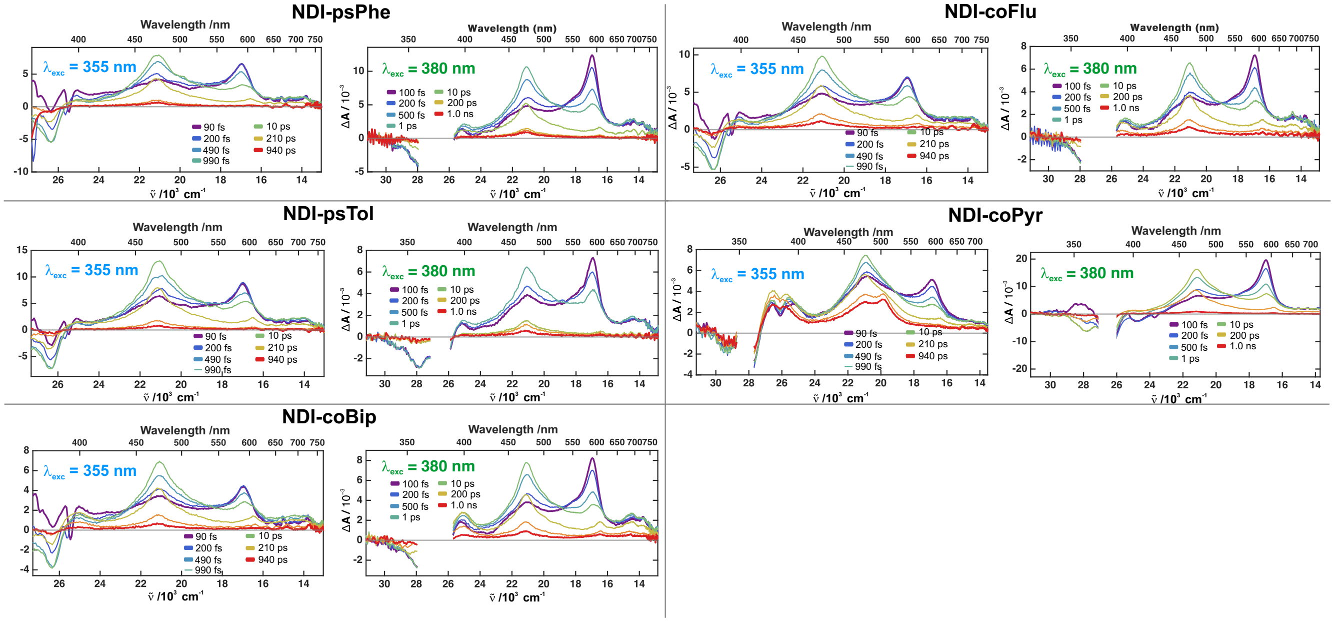

Figure S6 Transient absorption spectra measured after 355 nm excitation of the (co)polymers in DMF solutions.

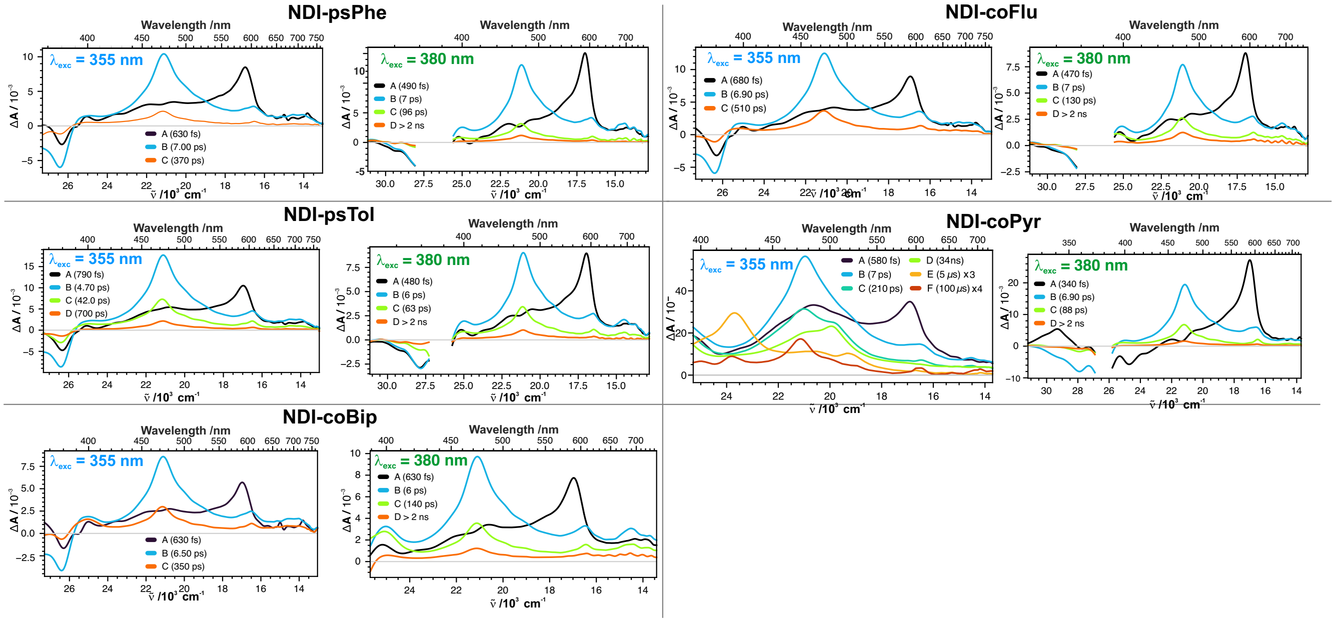

Figure S7 Evolution-associated difference absorption spectra and time constants obtained from a sequential global analysis with 3 (A $\rightarrow$ B $\rightarrow$ C $\rightarrow$), 4 (A $\rightarrow$ B $\rightarrow$ C $\rightarrow$ D $\rightarrow$) or 6 (A $\rightarrow$ B $\rightarrow$ C $\rightarrow$ D $\rightarrow$ E $\rightarrow$ F $\rightarrow$) steps of the TA data measured with the (co)polymer in DMF solution at various excitation wavelengths.

References

[1] S. Ye, T. Tian, A. J. Christofferson, S. Erikson, J. Jagielski, Z. Luo, S. Kumar, C.-J. Shih, J.-C. Leroux and Y. Bao, *Sci. Adv.*, 2021, **7**, eabd1794.

[2] S. Ye, N. Meftahi, I. Lyskov, T. Tian, R. Whitfield, S. Kumar, A. J. Christofferson, D. A. Winkler, C.-J. Shih, S. Russo, J.-C. Leroux and Y. Bao, *Chem*, 2023, **9**, 924–947.

[3] B. Lang, S. Mosquera-Vázquez, D. Lovy, P. Sherin, V. Markovic and E. Vauthey, *Rev. Sci. Instrum.*, 2013, **84**, 73107.

[4] R. J. Fernández-Terán, E. Sucre-Rosales, L. Echevarria and F. E. Hernández, *J. Chem. Educ.*, 2022, **99**, 2327–2337.

[5] S. Green and M. A. Fox, *J. Phys. Chem.*, 1995, **99**, 14752–14757.

[6] W. Zhao and F. N. Castellano, *J. Phys. Chem. A*, 2006, **110**, 11440–11445.

[7] J. F. Martinez, N. T. La Porte, C. M. Mauck and M. R. Wasielewski, *Faraday Discuss.*, 2017, **198**, 235–249.

[8] P. B. Merkel, P. Luo, J. P. Dinnocenzo and S. Farid, *J. Org. Chem.*, 2009, **74**, 5163–5173.

[9] P. Hapiot, C. Lagrost, F. Le Floch, E. Raoult and J. Rault-Berthelot, *Chem. Mater.*, 2005, **17**, 2003–2012.

[10] A. P. Davis and A. J. Fry, *J. Phys. Chem. A*, 2010, **114**, 12299–12304.

[11] A. Aster, C. Rumble, A.-B. Bornhof, H.-H. Huang, N. Sakai, T. Solomek, S. Matile and E. Vauthey, *Chem. Sci.*, 2021, **12**, 4908–4915.
